# Identifying Candidate Genes for Type 2 Diabetes Mellitus and Obesity through Gene Expression Profiling in Multiple Tissues or Cells

**DOI:** 10.1155/2013/970435

**Published:** 2013-12-26

**Authors:** Junhui Chen, Yuhuan Meng, Jinghui Zhou, Min Zhuo, Fei Ling, Yu Zhang, Hongli Du, Xiaoning Wang

**Affiliations:** ^1^School of Bioscience and Bioengineering, Guangdong Provincial Key Laboratory of Fermentation and Enzyme Engineering, South China University of Technology, Guangzhou 510006, China; ^2^Chinese PLA General Hospital, Beijing 100853, China; ^3^Guangdong Laboratory Animals Monitoring Institute, Guangzhou 510555, China

## Abstract

Type 2 Diabetes Mellitus (T2DM) and obesity have become increasingly prevalent in recent years. Recent studies have focused on identifying causal variations or candidate genes for obesity and T2DM via analysis of expression quantitative trait loci (eQTL) within a single tissue. T2DM and obesity are affected by comprehensive sets of genes in multiple tissues. In the current study, gene expression levels in multiple human tissues from GEO datasets were analyzed, and 21 candidate genes displaying high percentages of differential expression were filtered out. Specifically, *DENND1B*, *LYN*, *MRPL30*, *POC1B*, *PRKCB*, *RP4-655J12.3*, *HIBADH*, and *TMBIM4* were identified from the T2DM-control study, and *BCAT1*, *BMP2K*, *CSRNP2*, *MYNN*, *NCKAP5L*, *SAP30BP*, *SLC35B4*, *SP1*, *BAP1*, *GRB14*, *HSP90AB1*, *ITGA5*, and *TOMM5* were identified from the obesity-control study. The majority of these genes are known to be involved in T2DM and obesity. Therefore, analysis of gene expression in various tissues using GEO datasets may be an effective and feasible method to determine novel or causal genes associated with T2DM and obesity.

## 1. Introduction

T2DM, a complex endocrine and metabolic disorder, has become more prevalent in recent years, with significant adverse effects on human health. T2DM is characterized by insulin resistance (IR) and deficient *β*-cell function [1]. Interactions between multiple genetic and environmental factors are proposed to contribute to pathogenesis of the disease [1, 2]. Association of obesity with T2DM has been reported, both within and among different populations [3]. Earlier research has shown that obesity and its duration are major risk factors for T2DM, and IR pathological state generally exists in obesity [4, 5].

In recent years, numerous susceptibility loci have been identified through genome-wide association studies (GWAS) and meta-analyses on T2DM and obesity, and nearby candidate genes are proposed to be directly involved in the diseases [6, 7]. However, the underlying mechanisms by which these susceptibility loci affect and cause T2DM or obesity are currently unclear. Known SNPs associated with disease typically account for only a small fraction of overall disease [8, 9]. Gene expression patterns play a key role in determining pathogenesis and candidate genes of T2DM and obesity. A large-scale computable model has been created to analyze the molecular actions and effects of insulin on muscle gene expression [10]. Based on GWAS results, investigators integrated expression quantitative trait loci (eQTL) with coexpression networks to establish novel genes and networks relevant to the disease. Sixty-two candidate genes were identified through integrating 32 SNPs associated with T2DM and nearby gene expression from blood samples of 1008 morbidly obese patients. Many of the highly ranked genes are known to be involved in the regulation and metabolism of insulin, glucose, and lipids [11].

Different gene expression patterns exist in various tissues of organisms, and complex metabolic diseases, such as T2DM and obesity, are affected by comprehensive gene expression in multiple tissues. Analysis of gene expression in six tissues of mice from obesity-induced diabetes-resistant and diabetes-susceptible strains before and after the onset of diabetes led to the identification of 105 coexpression gene modules [12]. In the present study, gene expression profiles of human skeletal muscle, adipose tissue, islet, liver, blood and arterial tissue (or skeletal muscle, omental adipose tissue, cumulus cells, liver, blood, and subcutaneous abdominal adipose tissue) from GEO datasets were analyzed to identify the candidate genes for T2DM and obesity. Furthermore, candidate genes 1 Mb upstream and downstream (±1 Mb) of susceptibility SNPs for human T2DM and obesity were screened. Our analysis of gene expression in various tissues using GEO datasets provides a valuable method to determine novel candidate genes for T2DM and obesity.

## 2. Materials and Methods

**Figure 1:**
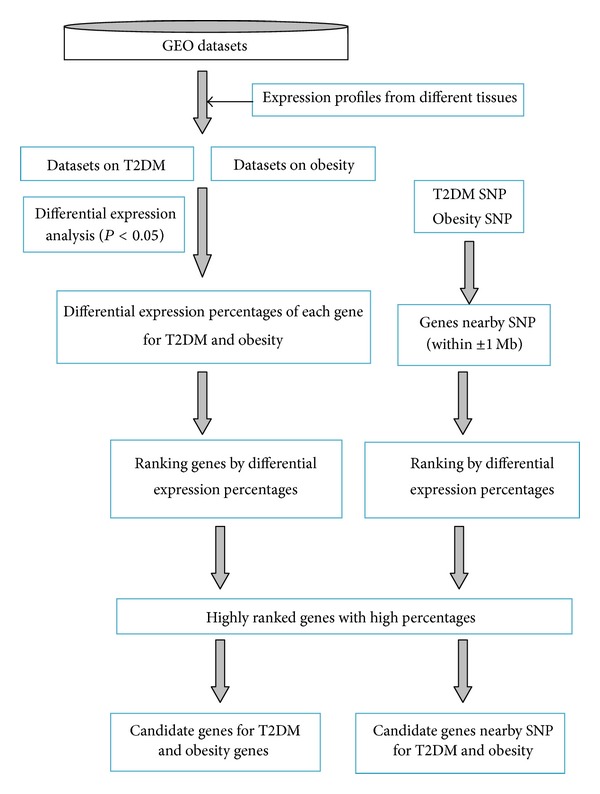
Overall experimental design.

The overall experimental design is shown in Figure 1.

### 2.1. GEO Dataset Selection and Statistical Analysis

Human GEO datasets for T2DM or obesity were downloaded from the Gene Expression Omnibus (GEO) database of NCBI (http://www.ncbi.nlm.nih.gov/gds/). In total, 23 datasets (14 for T2DM and 9 for obesity) were selected and downloaded. Some datasets were separated into several groups according to sample phenotype. Overall, 21 groups for T2DM and 14 for obesity were obtained. Samples of disease and control were included in the case and control subgroups, respectively (details of samples for each group are provided in Table S1 in Supplementary Material available online at http://dx.doi.org/10.1155/2013/970435). Three or more samples were included in each case or control subgroup for every microarray experiment. CEL files of samples were submitted to RMAExpress, Version 1.0.4, to yield normalized log_2_ expression values for each probe in individual groups with default parameters [13]. Analysis of variance (ANOVA) for normalized log_2_ expression values of two independent samples in each group was performed with the *F* test. The *t*-test for equal or unequal variances was used, depending on the *P*-value of the *F* tests.

Gene annotation files were downloaded from Ensembl (http://asia.ensembl.org/biomart/martview/45e0798c53bbd97ed0cf3d61142da3df) depending on the platform (GPL) of each group. Probes were matched with unique genes through gene annotation files. Probes corresponding to more than one gene were excluded. Probes or genes with significant differential expression were defined as *P*-value ≤ 0.05. We calculated the differential expression (*P* ≤ 0.05) percentage of each gene in all 21 T2DM and 14 obesity groups. For a gene with several probes, *P* values ≤ 0.05 were selected to represent significance.

### 2.2. Statistical Analysis of Differential Expression Percentages of Genes

Genes were ranked based on differential expression in the T2DM and obesity groups. Genes with the highest percentage of differential expression were identified as candidates (≥50% for T2DM and ≥60% for obesity). Ranked genes are presented in Supplementary Materials (Table S2).

### 2.3. Screening of Genes within ±1 Mb of Susceptibility SNPs for T2DM and Obesity

In total, 54 and 95 SNPs associated with T2DM and obesity, respectively, were selected (*P* ≤ 5 × 10^−8^, detailed information in Tables S3 and S4). The coordinate of each SNP in the chromosome was searched in the NCBI database (http://www.ncbi.nlm.nih.gov/SNP/). Consensus CDS (CCDS) files for human data were downloaded (ftp://ftp.ncbi.nlm.nih.gov/pub/CCDS/), and genes within ±1 Mb of SNPs were filtered out. Overall, 445 and 917 genes within 2 Mb of SNPs were associated with T2DM and obesity, respectively. The genes were reordered based on differential expression percentages with the above method, and those with the highest percentages were selected as candidates for T2DM (>40%) and obesity (>50%). Detailed information on all ranked genes in close proximity to SNPs is provided in Supplementary Materials (Table S5).

### 2.4. GO (Gene Ontology) and Pathway Analysis of Candidate Genes

Enrichment analysis of GO and pathways of all candidate genes was performed using Capital Bio Molecule Annotation System 3 (http://bioinfo.capitalbio.com/mas3/).

## 3. Results

### 3.1. Candidate Genes for T2DM and Obesity

In total, expression patterns of 23,810 genes were analyzed in the T2DM-control study. All genes were ranked based on the differential expression percentage. The average percentage of all genes was ~11%. Six highly ranked genes (*DENND1B*, *LYN*, *MRPL30*, *POC1B*, *PRKCB*, and* RP4-655J12.3*) were identified as candidates for T2DM (Table 1).

Since less groups were available for the obesity-control study, genes with fewer than 10 *P* values were excluded in order to obtain better statistical results. Expression of 14,367 genes was analyzed using the above method. The average percentage of all genes was ~17.5%. Eight genes (*BCAT1*, *BMP2 K*, *CSRNP2*, *MYNN*, *NCKAP5L*, *SAP30BP*, *SLC35B4*, and *SP1*) were isolated as candidates for obesity (Table 2).

### 3.2. Candidate Genes within ±1 Mb of SNPs Conferring Susceptibility to T2DM and Obesity

In total, 445 genes in close proximity to T2DM SNPs were reordered based on their differential expression percentages. In particular, two highly ranked genes,* HIBADH *and *TMBIM4*, within ±1 Mb of rs864745, rs849134, and rs1531343 SNPs were filtered out (Table 1).

Using the same method, seven highly ranked genes (*BAP1*, *GRB14*, *HSP90AB1*, *ITGA5*, *NCKAP5L*, *SP1*, and *TOMM5*) within ±1 Mb of obesity SNPs were identified (Table 2).

Gene symbols and the corresponding full names of all candidate genes are supplied in Tables 1 and 2.

### 3.3. GO and Pathway Analysis of Candidate Genes

**Figure 2:**
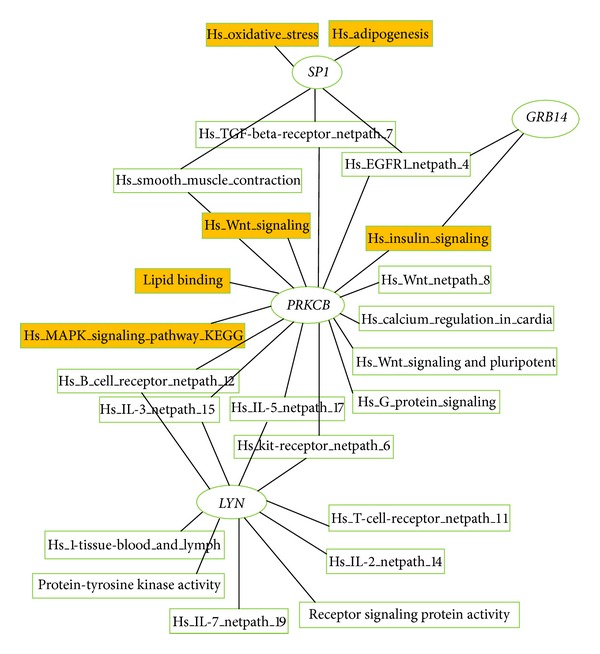
Enrichment analysis of GO and pathways for all genes. Yellow represents pathway directly or indirectly related to T2DM or obesity.

**Figure 3:**
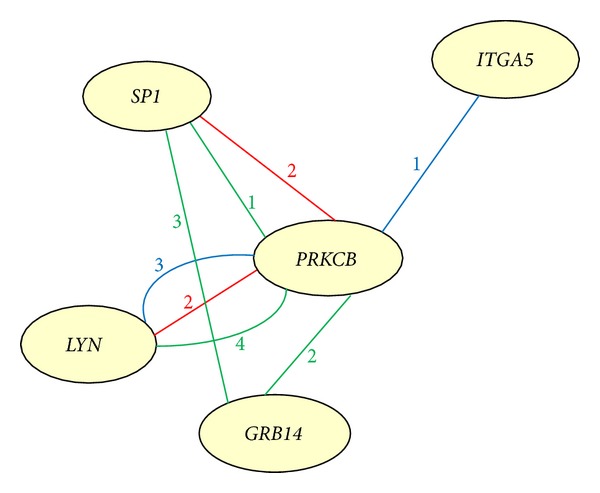
Correlation pathways of candidate genes. Red line, BioCarta; green line, GenMAPP; blue line, KEGG; number: counts of correlation pathways of two genes.

Results of GO and pathway analyses revealed that *PRKCB* is mainly associated with T2DM, and *PRKCB* and *GRB14 *are involved in insulin signaling within the gene pathway network (Figure 2). Further analysis of the correlation pathways of genes disclosed that *PRKCB*, *SP1*, *GRB14*, *LYN*, and *ITGA5* are correlated with each other (Figure 3).

## 4. Discussion

Complex metabolic diseases are often caused by alterations in gene expression or metabolic pathways in various tissues. Here, we analyzed differences in gene expression levels in various human tissues from GEO datasets in T2DM- or obesity-control experiments with the *t*-test. The *P* values were adjusted using the Bonferroni or FDR method [20] to allow for multiple testing. We introduced strict criteria with FDR ≤ 0.05. However, with these criteria, no genes were filtered out in most groups (16 of 21 groups, Table S6), while the percentage of genes with *t*-test *P* values ≤ 0.05 was lower than 10% in most groups (15 of 21 groups, Table S6). Therefore, the *t*-test statistic was ultimately applied for the present study. In total, we filtered out 21 candidate genes (8 for T2DM and 13 for obesity). The list of up- and downregulated candidate genes is provided in Supplementary Material (Table S7). Similarly, an eGWAS was performed across 130 independent experiments in human, rat, and mouse to identify additional genes implicated in the molecular pathogenesis of T2DM [21]. Interestingly, the same genes were not identified among the different studies. These discrepancies may be attributed to the use of various species, statistical methods, and tissues by different groups.

Analysis of the correlation pathways of the identified genes revealed that *PRKCB*, *SP1*, *GRB14*, *LYN*, and *ITGA5* are correlated with each other (Figures 2 and 3). The proteins interact directly within cells or indirectly among different tissues in the etiological process of T2DM or obesity. *PRKCB* mediates Ca^2+^ and DAG-evoked insulin secretion processes in Langerhans' *β* cells [22], functions downstream of insulin-receptor substrate 1 (IRS1) in muscle cells, and participates in the regulation of glucose transport in adipocytes by negatively modulating insulin-stimulated translocation of the glucose transporter, SLC2A4/GLUT4 [23, 24]. Under high glucose conditions in pancreatic beta cells, *PRKCB* may be involved in the inhibition of insulin gene transcription [25]. In the present study, we observed *PRKCB* upregulation in skeletal muscle, islets, adipose tissue, and blood and downregulation in liver of T2DM individuals (Table S7). These findings suggest that *PRKCB* may be involved in IR and deficient *β*-cell function *in vivo*. GRB14 binds directly to IR and regulates insulin-induced IR tyrosine phosphorylation [19]. *GRB14*-deficient mice display enhanced insulin signaling via IRS1 and AKT activation in liver and skeletal muscle, despite lower circulating insulin levels [18]. An earlier study showed increased *GRB14* expression in adipose tissues of both ob/ob mice and Goto-Kakizaki (GK) rats, but no changes in liver [26]. In our experiments, *GRB14 *expression was similarly increased in subcutaneous adipose tissue of obese humans, while a decrease was observed in liver (Table S7). In addition, *GRB14* is located within ±1 Mb of obese SNP rs10195252, and the rs10195252 T-allele is associated with increased *GRB14* subcutaneous adipose tissue mRNA expression [27]. However, *LYN* is implicated in the insulin signaling pathway via phosphorylation of IRS1 and PI3 K in liver and adipose tissues [14]. The insulin secretagogue, glimepiride, activates* LYN* in adipocytes [28]. This indirect *LYN* activation may modulate glycemic control activity of glimepiride in the extrapancreatic environment [28, 29]. In the present study, *LYN *expression was increased in adipose tissue, skeletal muscle, and blood of T2DM individuals, while a decrease was observed in islets and liver (Table S7). Moreover, *LYN* is a highly ranked gene with the highest differential expression percentage in the T2DM-control study (61.1%) and may therefore be a valuable candidate gene for future T2DM research. *ITGA5* additionally promotes PI3 K and AKT phosphorylation [30]. *ITGA5* expression was shown to be upregulated in adipose tissue of New Zealand obese (NZO) mice (high fat diet versus standard diet) [31]. We observed increased expression of *ITGA5* in human subcutaneous adipose tissue (Table S7). Moreover, *ITGA5 *is located within ±1 Mb of the obesity SNP, rs1443512. *SP1* is a zinc finger transcription factor that binds to GC-rich motifs and may be involved in insulin-mediated glucose uptake through positively regulating Glut4 expression in adipose tissue, skeletal muscle, and heart [32, 33]. *SP1* was downregulated in adipose tissue, while increased expression was observed in blood. Pathway analysis revealed the involvement of *SP1* in oxidative stress and adipogenesis (Figure 2). *SP1* not only is located within ±1 Mb of obesity SNP rs1443512 (similar to *ITGA5*), but also has the highest differential expression percentage (63.6%). Therefore, further studies are necessary to determine whether rs1443512 is related to *ITGA5* or *SP1* expression.

Differential expression of *HIBADH* ((+) 5.1*e* − 03) was reported in liver mitochondria during development of Goto-Kakizaki (GK) rats [15]. We observed no changes in *HIBADH* expression in liver, while a decrease was evident in skeletal muscle and blood of humans with T2DM. In addition, *HIBADH* is located within ±1 Mb of T2DM SNPs, rs864745, and rs849134. The association of *HIBADH* with T2DM requires further evaluation.An earlier study reported higher *BCAT1* expression in subcutaneous adipose tissue of females in the insulin-resistant than insulin-sensitive group [34]. Interestingly, higher* BCAT1* expression was observed in subcutaneous adipose tissue of obese humans in this study. We additionally recorded an increase in blood and decrease in omental adipose tissue (Table S7). *BCAT1* has been identified as the optimal marker for weight regain [35]. Moreover, the rs2242400 polymorphism in *BCAT1* appears to be associated with T2DM in more than one population [36]. *SLC35B4* has been identified as a potential regulator of obesity and insulin resistance in mouse models. Both *in vivo* and *in vitro* studies in mice disclosed that decreased *SLC35B4* expression is associated with a decrease in gluconeogenesis [17]. An increase in *SLC35B4* expression was observed in subcutaneous adipose tissue of obese humans in our study (Table S7). Interestingly, a SNP in the human *SLC35B4* gene (rs1619682) is associated with waist circumference [16]. *HSP90AB1* mRNA is reported to be upregulated in 3T3-L1 cells 6 h after stimulation of adipogenesis [37]. Moreover, *HSP90AB1* is located near the obesity SNP, rs6905288. Expression levels of *MYNN* are negatively correlated to BW (body weight) in adipose tissues of F2 mice (C57BL/6J × TALLYHO/JngJ) [38]. Consistently, our data showed that *MYNN* expression is downregulated in subcutaneous adipose of obese humans (Table S7). Furthermore, *SAP30BP *may be involved in body mass index (BMI) in adipose tissue (Pearson correlation (−0.51)) [39]. A decrease in *SAP30BP* expression was detected in subcutaneous adipocytes of obese human subjects in the present study (Table S7).

The rest of the candidates, *C2orf15*, *DENND1B*, *MRPL30*, *POC1B*, *RP4-655J12.3*, *TMBIM4*, *BMP2 K*, *CSRNP2*, *NCKAP5L*, *TOMM5*, and* BAP1*, may be novel genes related to T2DM or obesity. *TMBIM4 *is located within ±1 Mb of the SNP rs1531343 conferring susceptibility to T2DM, while *NCKAP5L*, *TOMM5*, and *BAP1* are mapped within ±1 Mb of SNPs conferring susceptibility to obesity. *TMBIM4* encodes transmembrane BAX inhibitor motif containing 4, which inhibits apoptosis induced by intrinsic and extrinsic stimuli and modulates both capacitative Ca^2+^ entry and inositol 1,4,5-trisphosphate (IP3)-mediated Ca^2+^ release [40]. In our study, *TMBIM4* was mainly upregulated in skeletal muscle, while downregulation was observed in liver (Table S7). The *NCKAP5L* gene encoding Nck-associated protein 5-like displayed upregulation in adipose tissue but was downregulated in blood (Table S7). *TOMM5* encodes the mitochondrial import receptor subunit TOM5 homolog. *TOMM5* was mainly involved in four GO terms (GO:0008565, protein transporter activity; GO:0015031, protein transport; GO:0005739, mitochondrion; GO:0005742, mitochondrial outer membrane). *BAP1* (ubiquitin carboxyl-terminal hydrolase) localizes at the nucleus and contains three domains (a ubiquitin carboxyl-terminal hydrolase (UCH) with an N-terminal catalytic domain, a unique linker region, and a C-terminal domain). The UCH domain conveys deubiquitinase activity to BAP1 [41]. In flies and humans, the Polycomb repressive deubiquitinase (PR-DUB) complex is formed through interactions of *BAP1* and ASXL1 [42]. *DENND1B* may promote the exchange of GDP with GTP and play a role in clathrin-mediated endocytosis [43]. The product of *MRPL30* is a constituent of mitochondrial ribosomes. *POC1B* is involved in the early steps of centriole duplication and the later steps of centriole length control [44, 45]. The *CSRNP2 *protein binds to the consensus sequence, 5′-AGAGTG-3′, and has a transcriptional activator. However, *C2orf15 *and* RP4-655J12.3 *have been rarely reported in databases or publications to date. Associations of all new candidate genes identified in the present study with obesity or T2DM require verification in future analyses.

## 5. Conclusions

*LYN*, a gene reported to be involved in the insulin pathway, was highly ranked with the highest differential expression percentage in the T2DM-control study (61.1%) and may therefore be a valuable candidate gene for future T2DM research. *NCKAP5L* with the highest differential expression percentage (63.6%) was located within ±1 Mb of the obesity susceptibility SNP, rs7132908, and was thus identified as the most likely novel candidate gene for obesity. We conclude that analysis of gene expression in various tissues via GEO datasets is an effective and feasible method to identify novel or causal genes associated with T2DM and obesity.

## Supplementary Material

Supplementary Table S1 Samples of each dataset studied in present study. Note; some datasets might be divided into two or more groups depending on sample phenotype.
Supplementary Table S2 All genes ranked by differential expression percentage.
Supplementary Table S3 Details about SNPs susceptibility to T2DM.
Supplementary Table S4 Details about SNPs susceptibility to obesity.
Supplementary Table S5 Ranked genes located within ± 1Mb of SNPs susceptibility to T2DM or obesity.
Supplementary Table S6 Comparison of p-values before and after adjusting by FDR.
Supplementary Table S7 Up- and down- expression of candidate genes in T2DM- and obesity-control study.

## Figures and Tables

**Table 1a:** (a) Highly ranked genes with high percentages in T2DM-control study

Gene symbol	Official full name	Location	Percentage	References
*LYN*	v-yes-1 Yamaguchi sarcoma viral related oncogene homolog	Chr8 56,792,386-56,925,006 (+): 8q13	61.1%	M$\ddot{\text{u}}$ller et al., 2000 [14]

*DENND1B*	DENN domain-containing protein 1B	Chr1 197,473,878-197,744,623 (−): 1q31.3	50%	

*MRPL30*	Mitochondrial ribosomal protein L30	Chr2 99,797,542-99,816,020 (+): 2q11.2	50%	

*POC1B*	POC1 centriolar protein homolog B (Chlamydomonas)	Chr12 89,813,495-89,920,039 (−): 12q21.33	50%	

*PRKCB*	Protein kinase C betatype PKC-beta (PKC-B)	Chr16 23,847,300-24,231,932 (+): 16p11.2	50%	Zhang et al., 2004 [22]

*RP4-655J12.3*		Chr1 116,916,755-116,917,283 (−): N/A	50%	

**Table 1b:** (b) Highly ranked genes nearby SNPs conferring susceptibility to T2DM

Gene symbol	Official full name	Location	Percentage	References	SNPs (Chr pos; frequencies)
*HIBADH*	3-Hydroxyisobutyrate dehydrogenase	Chr7 27,565,059-27,702,620 (−): 7p15.2	42.9%	Deng et al., 2010 [15]	rs864745 (28,180,556; *T* = 0.65, *C* = 0.35); rs849134 (28,196,222; *A* = 0.65, *G* = 0.35)

*TMBIM4*	Transmembrane BAX inhibitor motif containing 4	Chr12 66,530,717-66,563,807 (−): 12q14.1-q15	42.9%		rs1531343 (66,174,894; *G* = 0.81, *C* = 0.19)

Note: Chr pos: chromosome position; frequencies: the allele frequencies in 1000 genomes (http://www.ncbi.nlm.nih.gov/variation/tools/1000genomes/).

**Table 2a:** (a) Highly ranked genes with high percentages in obesity-control study

Gene symbol	Official full name	Location	Percentage	References
*BCAT1*	Branched chain amino-acid transaminase1	Chr12 24,962,958-25,102,393 (−): 12p12.1	63.6%	

*BMP2K*	BMP2 inducible kinase	Chr4 79,697,532-79,833,341 (+): 4q21.21	63.6%	

*CSRNP2*	Cysteine-serine-rich nuclear protein 2	Chr12 51,454,988-51,477,454 (−): 12q13.11-q13.12	63.6%	

*MYNN*	Myoneurin	Chr3 169,490,853-169,507,504 (+): 3q26.2	63.6%	Stewart et al., 2010 [38]

*NCKAP5L*	NCK-associated protein 5-like	Chr12 50,184,929-50,222,208 (−): 12q13.12	63.6%	

*SAP30BP*	SAP30 binding protein	Chr17 73,663,399-73,704,139 (+): 17q25.1	63.6%	Naukkarinen et al., 2010 [39]

*SLC35B4*	Solute carrier family 35, member B4	Chr7 133,974,089-134,001,827 (−): 7q33	63.6%	Fox et al., 2007 [16]; Yazbek et al., 2011 [17]

*SP1*	Sp1 transcription factor	Chr12 53,773,979-53,810,230 (+): 12q13.1	63.6%	

**Table 2b:** (b) Highly ranked genes nearby SNPs conferring susceptibility to obesity

Gene symbol	Official full name	Location	Percentage	References	SNPs (Chr pos; frequencies)
*NCKAP5L*	NCK-associated protein 5-like	Chr12 50,184,929-50,222,208 (−): 12q13.12	63.6%		rs7132908 (50,263,148; *G* = 0.73; *A* = 0.27)

*SP1*	Sp1 transcription factor	Chr12 53,773,979-53,810,230 (+): 12q13.12	63.6%		rs1443512 (54,342,684; *A* = 0.31; *C* = 0.69)

*ITGA5*	Integrin, alpha 5	Chr12 54,789,045-54,813,050 (−): 12q11-q13	58.3%		rs1443512 (54,342,684; *A* = 0.31; *C* = 0.69)

*TOMM5*	Translocase of outer mitochondrial membrane 5	Chr9 37,588,410-37,592,636 (−): 9p13.2	57.1%		rs16933812 (36,969,205; *G* = 0.40, *T* = 0.60)

*HSP90AB1*	Heat shock protein 90 kDa alpha (cytosolic), class B member 1	Chr6 44,214,849-44,221,614 (+): 6p12	55.6%		rs6905288 (43,758,873; *G* = 0.38, *A* = 0.62)

*BAP1*	BRCA1 associated protein-1	Chr3 52,435,024-52,444,009 (−): 3p21.31-p21.2	50%		rs6784615 (52,506,426; *C* = 0.04, *T* = 0.96)

*GRB14*	Growth factor receptor-bound protein 14	Chr2 165,349,323-165,478,360 (−): 2q22-q24	50%	Cooney et al., 2004 [18]; Holt et al., 2009. [19]	rs10195252 (165,513,091; *T* = 0.60, *C* = 0.40)

Note: Chr pos: chromosome position; frequencies: the allele frequencies in 1000 genomes (http://www.ncbi.nlm.nih.gov/variation/tools/1000genomes/).
