# Supplementary material for: Identifying Candidate Genes for Type 2 Diabetes Mellitus and Obesity through Gene Expression Profiling in Multiple Tissues or Cells
Source: J Diabetes Res. 2013 Dec 26;2013:970435. doi: 10.1155/2013/970435 (PMC3888709; doi:10.1155/2013/970435)
Supplement: Supplementary file 3 [file 970435.f3.doc]

**Table s3** **SNPs susceptibility to T2DM and nearby genes**.

| NO | SNP | Chr | Nearby Gene | Reference |
| --- | --- | --- | --- | --- |
| 1 | rs10923931 | 1 | NOTCH2 | Voight et al,2010 |
| 2 | rs7578597,rs11899863 | 2 | THADA | Zeggini et al,2008; Voight et al,2010 |
| 3 | rs243021 | 2 | BCL11A | Voight et al,2010 |
| 4 | rs2943641,rs7578326 | 2 | IRS1 | Voight et al,2010 |
| 5 | rs1801282,rs13081389 | 3 | PPARG | Chauhan et al,2010; Voight et al,2010 |
| 6 | rs4607103,rs6795735 | 3 | ADAMTS9 | Zeggini et al,2008; Voight et al,2010 |
| 7 | rs4402960,rs1470579 | 3 | IGF2BP2 | Saxena et al,2007; Scott et al,2007; Zeggini et al,2007; Voight et al,2010; Chauhan et al,2010 |
| 8 | rs10010131,rs1801214 | 4 | WFS1 | Voight et al,2010 |
| 9 | rs4457053 | 5 | ZBED3 | Voight et al,2010 |
| 10 | rs9465871,rs7754840,rs7756992,rs10946398,rs10440833 | 6 | CDKAL1 | WTCCC,2007; Saxena et al,2007; Scott et al,2007; Steinthorsdottir et al,2007; Zeggini et al,2007; Voight et al,2010; Chauhan et al,2010 |
| 11 | rs864745,rs849134 | 7 | JAZF1 | Zeggini et al,2008; Voight et al,2010 |
| 12 | rs972283 | 7 | KLF14 | Voight et al,2010 |
| 13 | rs896854 | 8 | TP53INP1 | Voight et al,2010 |
| 14 | rs13266634,rs3802177 | 8 | SLC30A8 | Sladek et al,2007; Voight et al,2010; Chauhan et al,2010 |
| 15 | rs10811661,rs10965250 | 9 | CDKN2A/B | Saxena et al,2007; Scott et al,2007; Zeggini et al,2007; Voight et al,2010; Chauhan et al,2010 |
| 16 | rs13292136 | 9 | CHCHD9 | Voight et al,2010 |
| 17 | rs12779790 | 10 | CDC123/CAMK1D | Zeggini et al,2008; Voight et al,2010; Chauhan et al,2010 |
| 18 | rs1111875,rs5015480 | 10 | HHEX/IDE | Zeggini et al,2007; Sladek et al,2007; Voight et al,2010; Chauhan et al,2010 |
| 19 | rs7903146 | 10 | TCF7L2 | Grant et al,2006; Helgason et al,2007; Chauhan et al,2010; Voight et al,2010 |
| 20 | rs2283228,rs2237895,rs2237897,rs231362,rs163184,rs2237892 | 11 | KCNQ1 | Yasuda et al, 2008; Unoki et al, 2008; Kong et al,2009; Voight et al,2010 |
| 21 | rs5215 | 11 | KCNJ11 | Chauhan et al,2010; Voight et al,2010 |
| 22 | rs1552224 | 11 | CENTD2 | Voight et al,2010 |
| 23 | rs10830963,rs1387153 | 11 | MTNR1B | Prokopenko et al,2009; Voight et al,2010 |
| 24 | rs1531343 | 12 | HMGA2 | Voight et al,2010 |
| 25 | rs7961581,rs4760790 | 12 | TSPAN8 | Zeggini et al,2008; Voight et al,2010 |
| 26 | rs7957197 | 12 | HNF1A | Voight et al,2010 |
| 27 | rs11634397 | 15 | ZFAND6 | Voight et al,2010 |
| 28 | rs8042680 | 15 | PRC1 | Voight et al,2010 |
| 29 | rs9939609,rs8050136,rs11642841 | 16 | FTO | WTCCC,2007; Zeggini et al,2007; Voight et al,2010 |
| 30 | rs757210,rs4430796 | 17 | HNF1B | Voight et al,2010 |
