# Supplementary material for: Identifying Candidate Genes for Type 2 Diabetes Mellitus and Obesity through Gene Expression Profiling in Multiple Tissues or Cells
Source: J Diabetes Res. 2013 Dec 26;2013:970435. doi: 10.1155/2013/970435 (PMC3888709; doi:10.1155/2013/970435)
Supplement: Supplementary file 4 [file 970435.f4.doc]

**Table s4 SNPs susceptibility to obesity and nearby genes.**

| NO | Nearest gene | SNP | Chr | Trait |
| --- | --- | --- | --- | --- |
| 1 | ADAMTS9 | rs6795735 | 3 | WHR adjusted for  BMI |
| 2 | BDNF | rs10767664 | 11 | BMI |
| 3 |  | rs4923461 | 11 | BMI |
| 4 |  | rs6265 | 11 | BMI |
| 5 |  | rs2030323 | 11 | BMI |
| 6 | C12orf51 | rs2074356 | 12 | WHR |
| 7 | CADM2 | rs13078807 | 3 | BMI |
| 8 | CDKAL1 | rs9356744 | 6 | BMI |
| 9 |  | rs2206734 | 6 | BMI |
| 10 | CPEB4 | rs6861681 | 5 | WHR adjusted for BMI |
| 11 | DNM3/PIGC | rs1011731 | 1 | BMI |
| 12 | ETV5 | rs9816226 | 3 | BMI |
| 13 |  | rs7647305 | 3 | BMI |
| 14 | FAIM2 | rs7138803 | 12 | BMI |
| 15 | FANCL | rs887912 | 2 | BMI |
| 16 | FLJ35779 | rs2112347 | 5 | BMI |
| 17 | FTO | rs1558902 | 16 | BMI/ WAIST |
| 18 |  | rs1121980 | 16 | BMI |
| 19 |  | rs9939609 | 16 | BMI/waist |
| 20 |  | rs8050136 | 16 | Fat percentage |
| 21 |  | rs17817449 | 16 | WAIST/ Extreme obesity |
| 22 |  | rs12149832 | 16 | WAIST |
| 23 | GNPDA2 | rs10938397 | 4 | BMI |
| 24 | GP2 | rs12597579 | 16 | BMI |
| 25 | GPRC5B | rs12444979 | 16 | BMI |
| 26 | GRB14 | rs10195252 | 2 | WHR adjusted for BMI |
| 27 | HOXC13 | rs1443512 | 12 | WHR adjusted for BMI |
| 28 | IRS1 | rs2943650 | 2 | Fat percentage |
| 29 | ITPR2/SSPN | rs718314 | 12 | WHR adjusted for BMI |
| 30 | KCTD15 | rs29941 | 19 | BMI |
| 31 |  | rs11084753 | 19 | BMI |
| 32 | KLF9 | rs11142387 | 9 | BMI |
| 33 | LRP1B | rs2890652 | 2 | BMI |
| 34 | LRRN6C | rs10968576 | 9 | BMI |
| 35 | LY86 | rs1294421 | 6 | WHR adjusted for BMI |
| 36 | LYPLAL1 | rs2605100 | 1 | WHR |
| 37 |  | rs4846567 | 1 | WHR adjusted for BMI |
| 38 | MAP2K5 | rs2241423 | 15 | BMI |
| 39 |  | rs4776970 | 15 | BMI |
| 40 | MC4R | rs571312 | 18 | BMI |
| 41 |  | rs17782313 | 18 | BMI/ Extreme obesity |
| 42 |  | rs6567160 | 18 | BMI |
| 43 |  | rs2331841 | 18 | BMI |
| 44 |  | rs12970134 | 18 | waist |
| 45 | MRPS22 | rs7638110 | 3 | BMI |
| 46 | MSRA | rs7826222 | 8 | WAIST |
| 47 | MTCH2 | rs3817334 | 11 | BMI |
| 48 |  | rs10838738 | 11 | BMI |
| 49 | MTIF3 | rs4771122 | 13 | BMI |
| 50 | NEGR1 | rs2815752 | 1 | BMI |
| 51 |  | rs2568958 | 1 | BMI |
| 52 | NFE2L3 | rs1055144 | 7 | WHR adjusted for  BMI |
| 53 | NISCH/STAB1 | rs6784615 | 3 | WHR adjusted for  BMI |
| 54 | NRXN3 | rs10150332 | 14 | BMI |
| 55 | NUDT3 | rs206936 | 6 | BMI |
| 56 | PAX5 | rs16933812 | 9 | Fat mass |
| 57 | PCSK1 | rs261967 | 5 | BMI |
| 68 | PRKD1 | rs11847697 | 14 | BMI |
| 59 | PTBP2 | rs1555543 | 1 | BMI |
| 60 | QPCTL/GIPR | rs2287019 | 19 | BMI |
| 61 |  | rs11671664 | 19 | BMI |
| 62 | POMC/ADCY3 | rs713586 | 2 | BMI |
| 63 |  | rs6545814 | 2 | BMI |
| 64 | RPL27A | rs4929949 | 11 | BMI |
| 65 | RSPO3 | rs9491696 | 6 | WHR adjusted for  BMI |
| 66 | SEC16B | rs543874 | 1 | BMI |
| 67 |  | rs574367 | 1 | BMI |
| 68 |  | rs516636 | 1 | BMI |
| 69 | SH2B1 | rs7359397 | 16 | BMI |
| 70 |  | rs7498665 | 16 | BMI |
| 71 | SLC39A8 | rs13107325 | 4 | BMI |
| 72 | SPRY2 | rs534870 | 13 | Fat percentage |
| 73 | TBX15/WARS2 | rs984222 | 1 | WHR adjusted for  BMI |
| 74 | TFAP2B | rs987237 | 6 | Waist/BMI |
| 75 | TMEM160 | rs3810291 | 19 | BMI |
| 76 | TMEM18 | rs2867125 | 2 | BMI |
| 77 |  | rs6548238 | 2 | BMI |
| 78 |  | rs7561317 | 2 | BMI |
| 79 | TNNI3K | rs1514175 | 1 | BMI |
| 80 | VEGFA | rs6905288 | 6 | WHR adjusted for  BMI |
| 81 | ZNF608 | rs4836133 | 5 | BMI |
| 82 | ZNF/KREMEN1 | rs4823006 | 22 | WHR adjusted for  BMI |
| 83 | BDNF | rs988712 | 11 | Extreme obesity |
| 84 | FAIM2 | rs7132908 | 12 | Extreme obesity |
| 85 | FTO | rs1421085 | 16 | Discovery in extreme  obesity + replication in population-based cohorts |
| 86 |  | rs9941349 | 16 | Extreme obesity |
| 87 |  | rs9936385 | 16 | Extreme obesity |
| 88 | KCNMA1 | rs2116830 | 10 | Extreme obesity |
| 89 | MAF | rs1424233 | 16 | Extreme obesity |
| 90 |  | rs8089364 | 19 | Extreme population/linear  regression BMI |
| 91 |  | rs17700144 | 18 | Extreme obesity |
| 92 | NRXN3 | rs11624704 | 14 | WHR in extreme population |
| 93 | TFAP2B | rs734597 | 6 | Discovery in extreme  obesity+ replication in population-based |
| 94 | MSRA | rs17150703 | 8 | Extreme obesity |
| 95 | TMEM18 | rs11127485 | 2 | Discovery in extreme  Obesity +replication in population-based |

**Note：rs7826222 has been merged into rs545854.**
